# Supplementary figures and images for: Obesity/Type II diabetes alters macrophage polarization resulting in a fibrotic tendon healing response
Source: PLoS One. 2017 Jul 7;12(7):e0181127. doi: 10.1371/journal.pone.0181127 (PMC5501654; doi:10.1371/journal.pone.0181127)

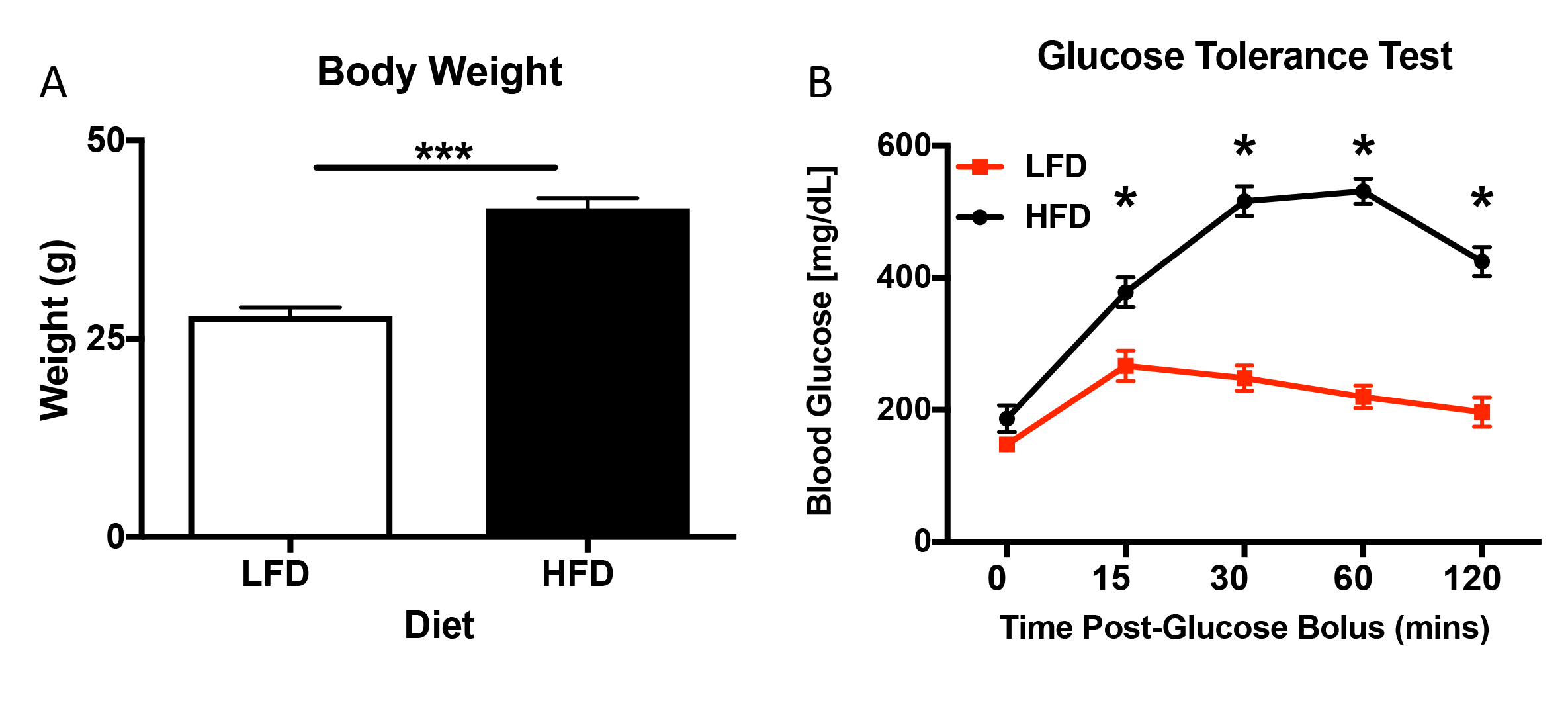

Supplement: S1 Fig — A—Body weights from MaFIA mice in both HFD (black bars) and LFD groups (white bars) were compared at the time of surgery, with HFD MaFIA mice weighing significantly more than LFD. C—Mice from the HFD MaFIA group (black line) demonstrated an impaired ability to respond to glucose loading relative to LFD MaFIA (red line), as shown by significantly elevated blood glucose levels at 15, 30, 60, and 120 minutes after delivering a glucose bolus. (*) Indicates p<0.05. (TIFF) [file pone.0181127.s001.tiff]

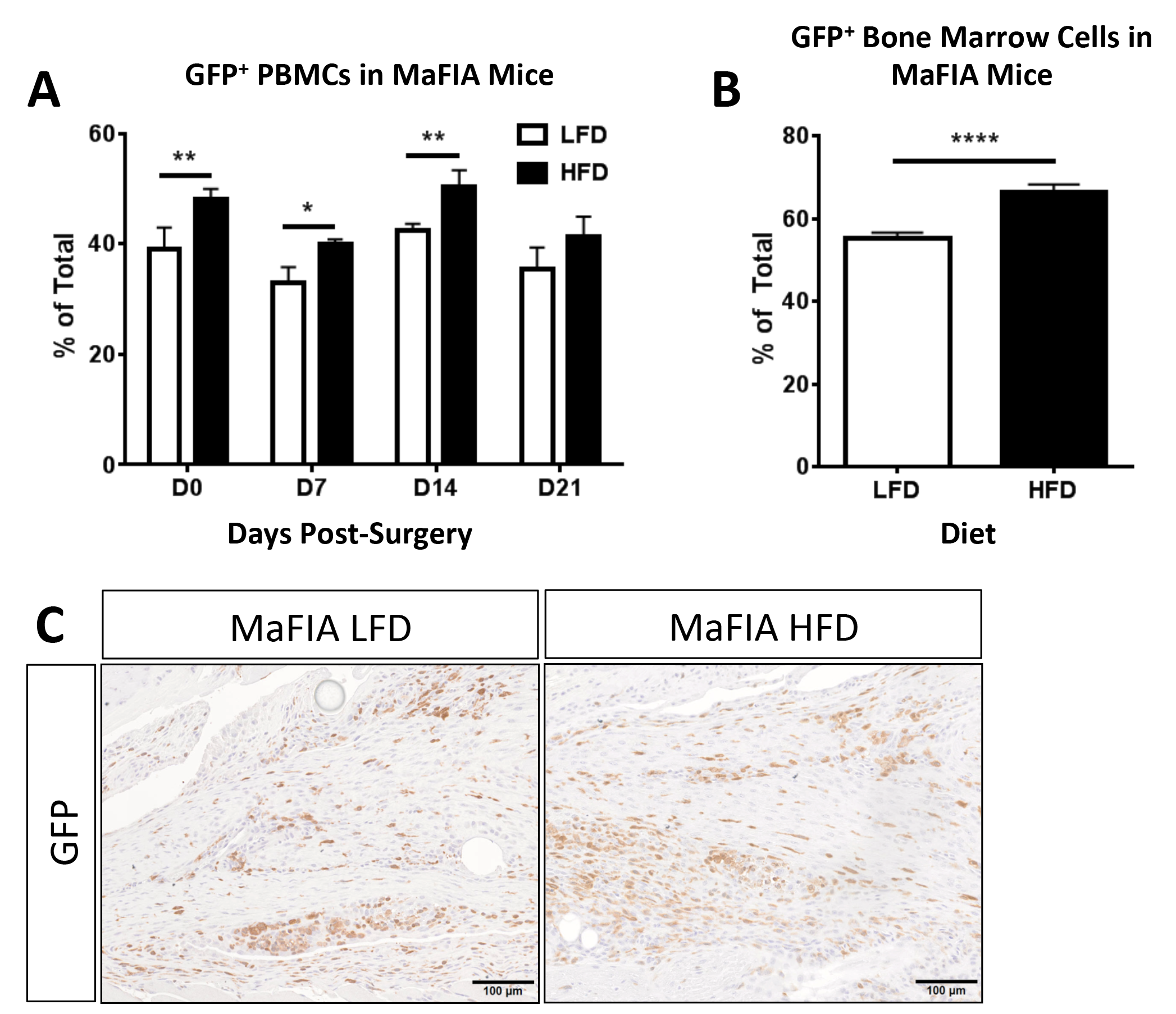

Supplement: S2 Fig — To assess changes in macrophage content in (A) peripheral blood mononuclear cells (PBMCs), (B) bone marrow, and (C) the healing flexor tendon, MaFIA mice, which express GFP in macrophages were used. Macrophage content was assessed via Flow cytometric analysis for GFP+ cells, in (A) Peripheral blood mononuclear cells (PBMCs) at days 0, 7, 14, and 21 days post-surgery, and (B) whole bone marrow at 21 days post-surgery. (*) Indicates p<0.05, (***) indicates p<0.0001 between HFD and LFD. No observable change in GFP expression was seen between LFD and HFD repairs, via GFP immunohistochemistry at day 21 post-surgery. Scale bar represents 100μm. (TIFF) [file pone.0181127.s002.tiff]
